# Force Generation by Molecular-Motor-Powered Microtubule Bundles; Implications for Neuronal Polarization and Growth

**DOI:** 10.3389/fncel.2015.00441

**Published:** 2015-11-10

**Authors:** Maximilian Jakobs, Kristian Franze, Assaf Zemel

**Affiliations:** ^1^Department of Physiology, Development and Neuroscience, University of CambridgeCambridge, UK; ^2^Bonn Cologne Graduate School of Physics and Astronomy, University of CologneCologne, Germany; ^3^Institute of Dental Sciences and Fritz Haber Research Center for Molecular Dynamics, Hebrew University of JerusalemJerusalem, Israel

**Keywords:** axon outgrowth, microtubules, molecular motors, force generation, neuronal polarization

## Abstract

The heavily cross-linked microtubule (MT) bundles found in neuronal processes play a central role in the initiation, growth and maturation of axons and dendrites; however, a quantitative understanding of their mechanical function is still lacking. We here developed computer simulations to investigate the dynamics of force generation in 1D bundles of MTs that are cross-linked and powered by molecular motors. The motion of filaments and the forces they exert are investigated as a function of the motor type (unipolar or bipolar), MT density and length, applied load, and motor connectivity. We demonstrate that only unipolar motors (e.g., kinesin-1) can provide the driving force for bundle expansion, while bipolar motors (e.g., kinesin-5) oppose it. The force generation capacity of the bundles is shown to depend sharply on the fraction of unipolar motors due to a percolation transition that must occur in the bundle. Scaling laws between bundle length, force, MT length and motor fraction are presented. In addition, we investigate the dynamics of growth in the presence of a constant influx of MTs. Beyond a short equilibration period, the bundles grow linearly in time. In this growth regime, the bundle extends as one mass forward with most filaments sliding with the growth velocity. The growth velocity is shown to be dictated by the inward flux of MTs, to inversely scale with the load and to be independent of the free velocity of the motors. These findings provide important molecular-level insights into the mechanical function of the MT cytoskeleton in normal axon growth and regeneration after injury.

## 1. Introduction

During development, neurons assume highly complex morphologies. After migration to their target location, neurons extend several short processes (neurites). One of these neurites eventually becomes an axon, the dominating cell process that can extend over considerable distances. The remaining neurites typically remain shorter and become highly branched dendrites. This change in cell morphology, so-called neuronal polarization, is critical for network formation and functioning. While many chemical signals controlling neurite growth and axon specification have been identified, the intracellular mechanisms driving outgrowth are still poorly understood (Suter and Miller, 2011).

A long-standing question pertains to the mechanism by which MTs and the actomyosin network, along the neurite and within the growth cone (GC), regulate axonal growth (Heidemann and Buxbaum, 1993; Suter and Miller, 2011; Dehmelt, 2014). The GC is the highly motile structure at the neurite tip which navigates the neurite to its target. Forces generated at the GC leading edge via actin polymerization and actomyosin contraction result in a (tensile) pulling stress on the emerging neurite (Lamoureux et al., 1989; Franze et al., 2009; Betz et al., 2011; Koch et al., 2012; Toriyama et al., 2013; Hyland et al., 2014). It has nevertheless been shown that neurites can grow and assume correct axonal morphologies even without a GC (Marsh and Letourneau, 1984; Letourneau et al., 1987; Ruthel and Hollenbeck, 2000). MTs accumulating next to the plasma membrane and later on pushed into the neurite shaft are believed to provide the complementary force needed for neurite initiation and growth (Ahmad and Baas, 1995; Dehmelt et al., 2006; Dehmelt, 2014). Within an emerging neurite as well as along the shaft of mature axons and dendrites, MTs are organized in thick and dense bundles comprising tens of filaments per cross-section, 1–100 μm in length, that are cross-linked by a variety of microtubule associated proteins (MAPs) including passive cross-linkers and molecular motors (Kapitein and Hoogenraad, 2015). A growing body of evidence indicates that the dynein and kinesin molecular motors residing in these bundles exert sliding forces between the MTs, which push against the actomyosin cortex that surrounds those bundles (Ahmad et al., 2000; Dehmelt et al., 2006; Jolly et al., 2010; Lu et al., 2013, 2015; Roossien et al., 2014). Although MTs eventually undergo ‘catastrophe’ and depolymerize under load, experiments show that they could still bear significant compressional forces before they do so and thus contribute to the outward pushing of obstacles (Janson et al., 2003). A well known example is their role in the assembly and dynamics of the mitotic spindle during cell division (Civelekoglu-Scholey and Scholey, 2010; Mogilner and Craig, 2010). There is also ample of evidences that MT pushing forces are involved in axon initiation and growth. Both the depletion of MTs from the core of axons and the inhibition of cytoplasmic dynein or kinesin-1 motors result in axon retraction and impaired growth, while dismantling of actin filaments or inhibition of myosin II molecular motors facilitates growth (Bradke and Dotti, 1999; Ahmad et al., 2000; Dehmelt et al., 2006; Jolly et al., 2010; Lu et al., 2013, 2015; Roossien et al., 2014).

Additionally, the tension along neurites was shown to increase with MT depolymerization and decrease with actin disruption (Dennerll et al., 1988). Forces on the order of 10^2^–10^3^ pN have been reported to be essential for mechanically initiating axon growth by external loading (Bray, 1984; Dennerll et al., 1989; Zheng et al., 1991; Chada et al., 1997; Fass and Odde, 2003). These experiments provide estimates of the restoring forces that may be acting on the MT bundles in developing axons and dendrites. This range of forces is approximately 10–100-fold higher than the force needed to pull a tether from a (bare) lipid bilayer (Dai and Sheetz, 1995; Hochmuth et al., 1996; Atilgan et al., 2006). The larger range of resisting forces is believed to arise from the cytoskeletal cortex underlying the lipid bilayer and the contractile actomyosin forces generated therein (Ahmad et al., 2000; Xu et al., 2013).

These observations highlight the role of molecular motor activity in the regulation of axon initiation and growth. There is nevertheless poor understanding of how molecular motors cooperatively function in the dense MT bundles of axons and dendrites. While some processes, such as filament sorting and bundle expansion, have been investigated (Kapitein et al., 2005, 2008; Kerssemakers et al., 2006; Braun et al., 2009), experimentally monitoring the molecular organization and motion of single filaments within neurites remains challenging. Computer simulations provide an invaluable complementary tool to gain molecular-level insight into the internal organization, dynamics and function of these cellular structures. They also provide the opportunity to dissect the contribution of individual molecular constituents to the overall macroscopic behavior of the system and determine the most crucial parameters for force generation. Models of both actomyosin and MT bundles have been reported and studied in different contexts such as stress fiber formation and cytokinesis (Nédélec, 2002; Mogilner et al., 2006; Paul et al., 2009; Lenz et al., 2012; Kim, 2014; Bidone et al., 2015; Ward et al., 2015).

In this manuscript, we present a computational investigation of the dynamics and force generation properties of 1D bundles of MTs that are cross-linked and powered by molecular motors. The dynamics of bundle expansion are analyzed as a function of the motor type, bundle polarity, filament length, filament-motor connectivity, and strength of the applied load. We demonstrate that both unipolar and bipolar motor types (e.g., kinesin-1 and kinesin-5, respectively) efficiently sort oppositely oriented filaments. However, only unipolar motors are found to provide the driving force for bundle expansion, while bipolar motors hinder this motion. The capacity of MT bundles to exert a force is shown to depend sharply on the fraction of cross-links formed by unipolar motors; only if a threshold fraction is surpassed can the bundle become percolated and exert a force on the boundaries. The dependence of this percolation threshold on the MT density and length is presented. We also investigated the growth of MT bundles in the presence of a constant influx of MTs. Beyond a short equilibration period the bundles grow linearly in time. In this (steady-state) growth regime, the growth velocity is found to be dictated by the inward flux of MTs and the magnitude of the opposing load, but interestingly, to be independent of the free velocity of the motors. This is because at this stage of growth most filaments slide with the growth velocity while the relative velocity between filaments is small and the force exerted by the motors approaches the maximal stall force of the motors; similar behavior has been observed experimentally (Suter and Miller, 2011; Roossien et al., 2014). The bundle width and growth velocity adjust spontaneously to the load. We discuss these findings in the context of neurite initiation and growth and their implications for axon specification.

## 2. Model

### 2.1. Bundle structure

**Figure 1:**
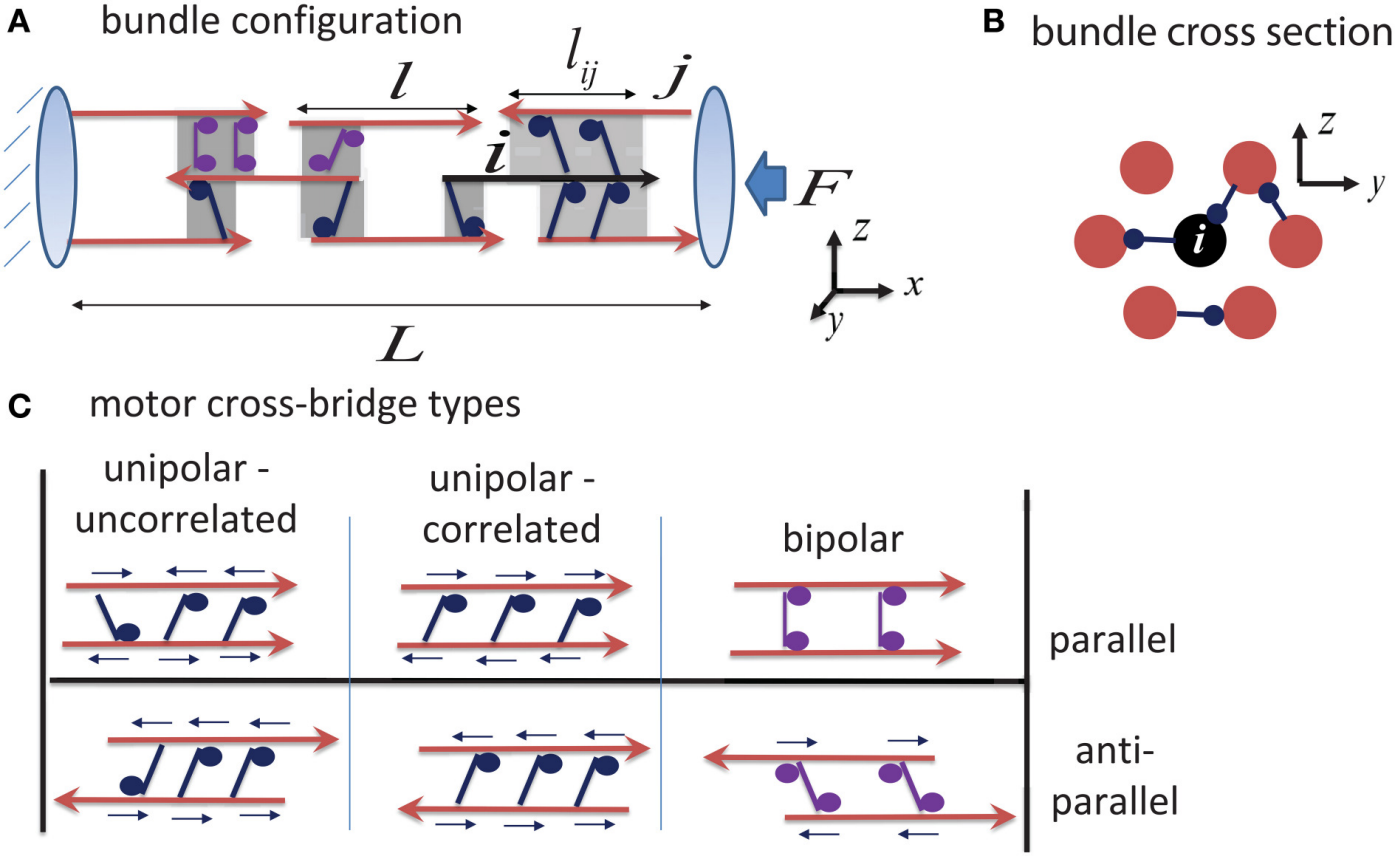
**Schematic illustration of bundle structure. (A)** Shows a side view of the bundle connectivity. MT filaments are shown as red arrows (indicating the MT polarity), and shaded regions mark the overlap regions between them which may either be cross-linked by motors, or be unoccupied. Highlighted in black is a “central filament,” *i*, interacting with its neighboring filaments. The force on any filament *i* is calculated by summing the contributions from all motors that interact with it (see Equation 1); the calculation uses the overlap length between the filaments, *l*_*ij*_, that is assumed to be proportional to the number of motors cross-linking the filaments. The left boundary is held fixed (mimicking the dense cytoskeleton at the neurite entry), and expansion occurs to the right against an opposing load, *F*, arising from the contractile actomyosin cytoskeleton in the neurite cortex. **(B)** Shows the hexagonal packing of filaments in a bundle cross-section (Chen et al., 1992). Each filament potentially has six nearest neighbors in a cross section, but it may interact with more than six filaments along its length. **(C)** Illustrates three distinct motor-filament configurations; see Section 2.2 for the consequences of these interaction types. Small arrows above each motor indicate the direction of the force that the motor exerts on the filaments. Note how bipolar motors fail to exert forces between parallel filaments.

We consider a cylindrical bundle of MTs, cross-linked by ensembles of molecular motors and oriented along the *x*-axis of a Cartesian coordinate system (Figure 1). The bundle comprises a total of *N* = *N*_*R*_ + *N*_*L*_ filaments of which *N*_*R*_ point with their + end to the right (assigned as the GC direction) and *N*_*L*_ point with their + end to the left (cell body direction). For simplicity we assume that all filaments in a bundle have an equal length, *l*. The filaments are arranged on a hexagonal lattice in the *y* − *z* plane and along the bundle axis. We set the initial bundle length to *L*_0_, and sequentially position the *N* filaments along the *x*-axis with their centers randomly chosen between *x* = 0 to *x* = *L*_0_ and their *y, z* coordinates increasing gradually from the bundle center outward to obtain a hexagonally packed cylindrical bundle (Figure 1B). The inter-filament spacing in the *y* − *z* plane (~50 nm) is assumed to allow individual molecular motors to intervene between the filaments and cross-link them with their respective “cargo” or “walking” domains. This architecture allows the motors to slide the filaments past each other and to collectively induce global changes in bundle length and force (Chen et al., 1992; Kapitein et al., 2005, 2008; Kerssemakers et al., 2006; Braun et al., 2009).

Axons and dendrites comprise different types of motor proteins that may generally be classified as unipolar or bipolar motors (Zemel and Mogilner, 2009). Unipolar motors, such as cytoplasmic dynein or kinesin-1, possess one “walking” domain with which it preforms a power-stroke and moves along a filament, and one “cargo” domain, with which it binds a cargo or another filament. Bipolar motors, such as kinesin-5, have walking domains on both ends.

Only very little is known on how these motors organize between adjacent MTs in dense bundles such as those of axons and dendrites. It is generally expected that some degree of cooperativity exists in the binding of the motors to the filaments and that this will render the motors to locally segregate between the filaments. As a working hypothesis, we thus assume that each overlapping region between filaments is occupied by one type of motor only, or is otherwise devoid of motors. This approximates the more complex situation that may exist where different motors occupy the same region between filaments and one motor type dominates due to inter-motor binding correlations. We distinguish two generic binding arrangements of unipolar motors to neighboring filaments (left panels Figure 1C). In one scenario, the motors bind the filaments in an uncorrelated fashion with their cargo or walking domains oriented randomly between the filaments; in a second scenario, they bind the filaments in a correlated fashion having their cargo and walking domains oriented toward the same MT.

During bundle configuration, all possible overlap regions between filaments are identified and their probability of being cross-linked by unipolar or bipolar motors is determined by a control parameter, χ_*i*_, which dictates the fraction of MT overlaps that are cross-linked by the given motor type *i* = {*up, bp*}. The total fraction of overlaps that are populated by motors is given by χ_*up*_ + χ_*bp*_ = χ. In the calculations presented below we typically assumed that χ = 1, indicating that all overlapping regions between filaments are cross-linked by motors; as would be the case for sufficiently high density of motors in the bulk. We nevertheless devote a whole Section (3.2) to demonstrate the interesting role that χ may have in dilute motor systems.

During the simulation, filaments are sled by the motors they connect to according to force-velocity relations which we detail below. It is assumed that the motors maintain their interaction to the filaments as long as the overlap between them exists. Once an overlap is broken, the motors are assumed to bind elsewhere in the bundle such that the proportion between motor types χ_*i*_ remains fixed. The use of χ_*i*_, although simplifies the much more complicated thermodynamic problem of considering the energetic tendency of the motors to populate the overlap regions between filaments, allows us to draw important conclusions on how a mixture of motors may affect the forces that such bundles may exert.

### 2.2. Model equations

We here derive the equations used in our simulations to calculate the motion of filaments in the bundle. Because the motion of MTs in the dense bundles we discuss is essentially one-dimensional we only consider filament movements along the *x*-axis and neglect transverse (radial) forces that may arise due to interactions with the lipid membrane or membrane-associated proteins. In addition, in the low Reynolds number environment of the cell the velocity of each filament is proportional to the total force exerted on it (Howard, 2001). We thus write the following equation for the velocity of each filament, *i*, in the bundle :

(1) $$\xi \;v_{i}=f_{i}=\sum_{j}f_{i,j}^{m}+f_{i}^{ex}$$

The first term on the right hand side (rhs), $f_{i}^{m}=\underset{j}{\sum }f_{i,j}^{m}$, is the force exerted on filament *i* due to its motor-driven interaction with all nearby filaments *j* (see Figure 1A). Each contribution, $f_{i,j}^{m}=f_{i,j}^{m}\left(v_{j}-v_{i}\right)$, depends on the relative velocity of the two interacting filaments; the specific form of this force-velocity relation is dictated by the type of motors and their organization in between the filaments, as discussed below. For simplicity, linear force-velocity relationships have been assumed; this is consistent with experiments (Mallik et al., 2004; Valentine et al., 2006), and generalization of this approach to more complex force-velocity relationships is numerically straightforward. The second term is the contribution of external forces; in our simulations it is applicable only to filaments in contact with the boundaries. On the left, ξ, is the drag coefficient representing the viscous environment of filaments in the bundle. We anticipate that ξ is significantly higher than the drag coefficient in aqueous solution due to weak binding interactions of the filaments with other proteins in their neighborhood; we used the value ξ = 0.023 pN s/μm per 1 μm filament based on diffusion constant measurements reported in Tawada and Sekimoto (1991).

#### 2.2.1. Motor cross-bridging types and corresponding force-velocity relations

We assume that the motors occupy the overlapping regions between neighboring filaments with a uniform and constant density, λ, and that the force they exert is additive and proportional to the total overlap length *l*_*ij*_ of neighboring filaments, see Figure 1A. Consequently, λ*l*_*ij*_, is the mean number of motors within this overlap. For simplicity, we assume that motors along *l*_*ij*_ share the load equally and omit the complex non-linear effect of indirect non-linear inter-motor interactions (Klumpp and Lipowsky, 2005; Kunwar et al., 2008). The forces generated by the motors are characterized by force-velocity relationships which we specify below for the different possible cross-bridging types.

##### 2.2.1.1. Orientationally correlated unipolar motors

In an overlap region between filaments, unipolar motors may be correlated, or randomly oriented as illustrated Figure 1C. In the former case, all motors in a given overlap bind their walking domains to one filament and their cargo domains to the other filament. While not much is known about the orientation of motors in the crowded environment of dense MT bundles, some indirect evidence for such arrangement of motors exists (Haimo and Rosenbaum, 1981; Haimo and Fenton, 1984; Vilfan et al., 2001; Sciambi et al., 2005). In a dense interconnected bundle, each MT filament may interact with multiple MTs at the same time (Figures 1A,B) and the motors within each overlap region can exert a different net force on the given filament. When expressing the force acting on a given filament, *i*, it is essential to know if the motors in an overlapping region with a filament *j* are bound with their walking domains to filament *i* or to filament *j*. The following force-velocity relationship holds when motors bind their walking domains to filament *i* and cargo domains to filament *j*:

(2) $$f_{i,j}^{m}=-\lambda l_{ij}f_{s}\left[n_{i}-\frac{v_{j}-v_{i}}{v_{0}}\right];$$

when the motors' walking domains are bound to filament *j* one has:

(3) $$f_{i,j}^{m}=\lambda \;f_{s}\;l_{ij}\;\left[n_{j}-\frac{v_{i}-v_{j}}{v_{0}}\right]$$

The factors *f*_*s*_ and *v*_0_ are the stall force and free velocity of the motors, respectively. $n_{i}\hat{x}$ is the walking direction of the molecular motor on the filament, e.g., a value of *n*_*i*_ = −1 represents a motor that walks in the negative direction of the *x*-axis (for instance when a minus-end directed motor moves on a filament that points with its minus-end toward the negative direction of the *x*-axis).

##### 2.2.1.2. Orientationally uncorrelated unipolar motors

When the binding of unipolar motor proteins to a pair of neighboring filaments is orientationally uncorrelated, we sum the contributions of the two populations of motors in that overlap region; this leads to the following force-velocity relationship in the limit of large numbers of motors:

(4) $$f_{i,j}^{m}=\frac{1}{2}\;\lambda \;f_{s}\;l_{ij}\;\left[(n_{j}-n_{i})+\frac{2(v_{j}-v_{i})}{v_{0}}\right]$$

The factor 1∕2 reflects the equal probability of individual motors to bind the filaments in a given overlap with either their walking or cargo domains. Note that for an isolated parallel pair (i.e., *n*_*j*_ = *n*_*i*_ and no other cross-links), Equations (4) and (1) yield *v*_*i*_ = *v*_*j*_ = 0, as expected, because the motor forces on each filament cancel each other. This is in contrast to the correlated motor binding case, in which the motors can exert significant sliding forces on a pair of parallel filaments. For an antiparallel pair, Equations (4) and (2) are equivalent.

##### 2.2.1.3. Bipolar motors

The situation in this case is similar to that of uncorrelated unipolar motors, if we assume that the two walking domains of each motor are independent of one another. Unlike unipolar motors, a bipolar motor can glide between the filaments with a finite velocity, ${\vec{v}}_{m}=v_{m}\hat{x}$, relative to a stationary frame of reference. If this motor is connected to a pair of filaments {*i*,*j*}, it exerts equal and opposite forces *f*_*i*_ = −*f*_*j*_. Implementing a linear force-velocity relationship for each of the walking domains independently yields: *f*_*i*_ = −*f*_*s*_[*n*_*i*_ − (*v*_*m*_ − *v*_*i*_)∕*v*_0_] and *f*_*j*_ = −*f*_*s*_[*n*_*j*_ − (*v*_*m*_ − *v*_*j*_)∕*v*_0_]. We thus find a limiting equation for the motor velocity in a pair of sliding filaments: $v_{m}=\frac{1}{2}\left[v_{i}+v_{j}+v_{0}\left(n_{i}+n_{j}\right)\right]$. Substituting this in the expression for *f*_*i*_ we find:

(5) $$f_{i,j}^{m}=\frac{1}{2}\lambda f_{s}l_{ij}\left[(n_{j}-n_{i})+\frac{v_{j}-v_{i}}{v_{0}}\right]$$

Note that the only apparent difference between this case and Equation (4) above is that bipolar motors slide the filaments with free velocity that is twice as large as that driven by orientationally uncorrelated unipolar motors. In addition, bipolar motors that distribute uniformly between two *parallel* filaments do not exert a net force on the filaments but merely bundle them together or act as viscous elements that slow down their relative motion. This applies to motors that persistently move across the filaments with constant speed, as observed for instance for the bipolar motor kinesin-5 (Cheerambathur et al., 2008; Kapitein et al., 2008). Because orientationally uncorrelated unipolar motors behave similarly to bipolar motors we discuss them interchangeably in what follows and draw the comparison between correlated-unipolar motors and bipolar motors.

Within an interconnected bundle each filament, *i*, may have multiple cross-links with other filaments and the cross-bridges may be of the different types discussed above; the overall motor-generated force is calculated as a sum: $f_{i}^{m}=\underset{j}{\sum }f_{i,j}^{m}$. This, together with the boundary conditions discussed below, constitute an algebraic set of linear equations for the velocities of all the filaments in the bundle. Once the velocities have been determined, all filaments are propagated by a small distance, *v*_*i*_ *dt*, where *dt* ~ 0.2 s is a sufficiently small time step to avoid numerical error. This results in a “trajectory” of the filaments in the bundle that can be averaged over an ensemble of starting configurations. Between 50 and 1000 trajectories have been averaged to characterize the mean behavior of the bundles. For simplicity, all bundles studied comprised either plus-end or minus-end-directed motors but not their mixtures.

#### 2.2.2. Boundary conditions

In all simulations an opposing force, *F*^*ex*^, was assumed to act on the right boundary to resist the motor-driven motion of the filaments. The left boundary was assumed to be supported by a stiff spring that sustains the same load but in the opposite direction. We considered two types of external load: (i) a fixed load, *F*^*ex*^ = const, (ii) an elastic load, $F^{ex}\left(t\right)=-k\left[L\left(t\right)-L_{0}\right]$, where *k* is the spring constant of the compliant right boundary. In either case, the total force *F*^*ex*^ distributes among all filaments touching the right boundary, and −*F*^*ex*^ distributes over the filaments touching the left boundary. The distribution of *F*^*ex*^ and −*F*^*ex*^ amongst the different filaments on the right and left boundaries, respectively, is not necessarily even. Rather, we assumed that all the right boundary filaments, whose tips exceed the boundary *x* = *L*(*t*), displace with equal speed, *v*_*R*_; and all filaments exceeding the left boundary, *x* = 0, (principally) move with equal speed *v*_*L*_. These conditions are formulated as follows: For the right boundary filaments:

(6) $$\sum_{i\;=\;1}^{n_{R}}f_{i}^{ex}=F^{ex}$$

(7) $$v_{1}=v_{2}=\cdot \cdot \cdot =v_{R}$$

For the left boundary filaments:

(8) $$\sum_{i=1}^{n_{L}}f_{i}^{ex}=-F^{ex}$$

(9) $$v_{1}=v_{2}=\cdot \cdot \cdot =v_{L}$$

In our simulations, we used a stiff spring on the left boundary to prevent motion of that boundary; hence, *v*_*L*_ ≈ 0, and the speed of bundle expansion is given by *dL*∕*dt* = *v*_*R*_. These conditions supplement the set of equations, Equation (1), for the velocities of the filaments, with *n*_*R*_ + 1 new equations to solve for the *n*_*R*_ + 1 unknowns, $\left\{f_{i}^{ex}\right\}$ and *v*_*R*_, on the right boundary, and *n*_*L*_ + 1 new equations for the *n*_*L*_ + 1 unknowns, $\left\{f_{i}^{ex}\right\}$ and *v*_*L*_ on the left boundary. For all other filaments $f_{i}^{ex}$ is identically zero. For *N* filaments one diagonalizes an *N* × *N* matrix at each iteration in time to solve for the velocities of all filaments in the bundle.

## 3. Results

### 3.1. Unipolar motors provide the driving force for bundle expansion and bipolar motors hinder it

Bundles comprising a total of *N* ~ 100 MTs (of which *N*_*R*_ and *N*_*L*_ point with their plus-ends to the right and left, respectively), closely packed in a hexagonal array within a cylinder of axial length *L*_0_ = 50 μm, were simulated subject to an opposing spring on the right boundary and a fixed boundary on the left; the total force generated by the bundle is *F*(*t*) = *k*[*L*(*t*) − *L*_0_], where *L*(*t*) is the evolving bundle length and *k* is the external spring constant.

**Figure 2:**
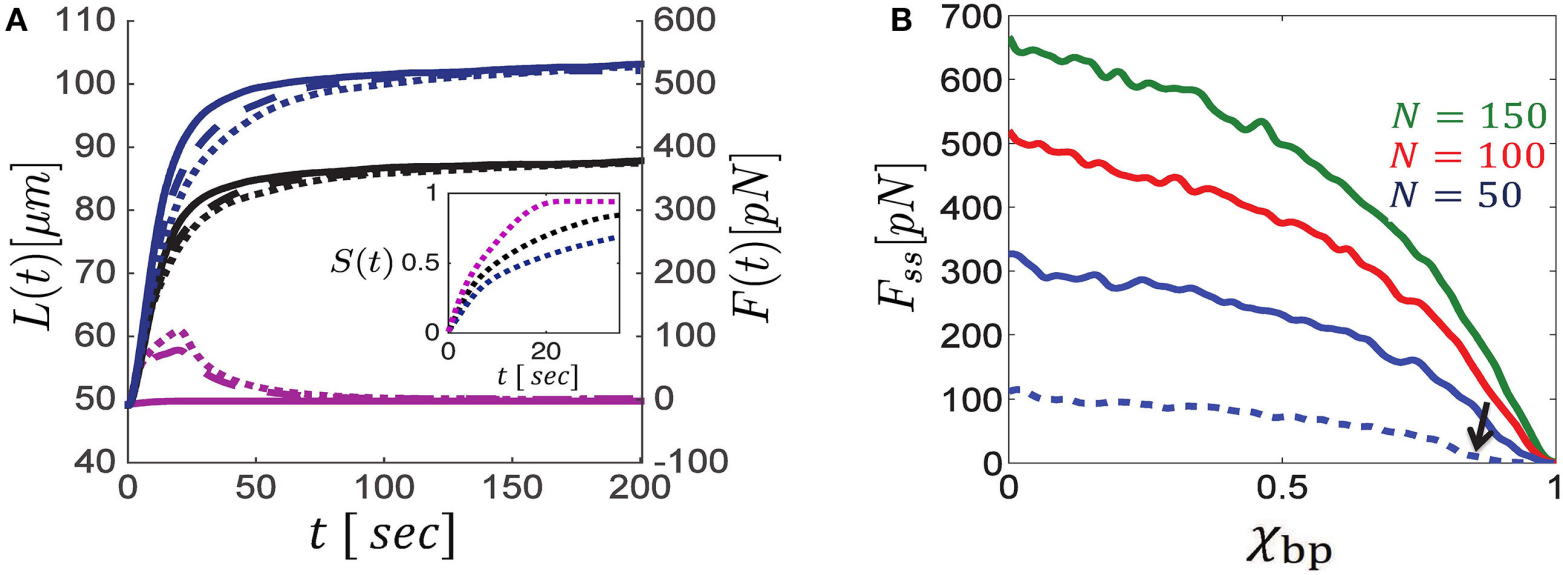
**Dynamics of bundle expansion and force generation, shown for varying polarity ratios of filaments and fraction of bipolar cross-bridges. (A)** Shows the evolution of bundle length (left *y*-axis) and force (right *y*-axis) for different fractions of bipolar cross-bridges, χ_*bp*_, and different polarity ratios, *N*_*R*_∕*N*; the number of filaments is *N* = 100. Blue curves correspond to bundles comprising unipolar cross-bridges only (χ_*bp*_ = 0), black curves are for a 50:50 mixture of bipolar and unipolar cross-bridges (χ_*bp*_ = 0.5), and purple curves are for bundles with bipolar cross-bridges only (χ_*bp*_ = 1). Solid, dashed and dotted curves correspond to polarity ratios: *N*_*R*_∕*N* = 1, 0.2, 0.5, respectively. Bundle polarity is found to have a minor effect on the expansion dynamics and no effect on the steady state force. The inset shows the dynamics of filament sorting in mixed bundles (*N*_*R*_∕*N* = 0.5) as quantified by the order parameter *S*(*t*) (defined in the text); *S* = 0 is found for a bundle that is locally mixed across its length, and *S* = 1 is found for a bundle that is locally parallel all along. *S*(*t*) evolves to ~1 on a time scale of ~10 s, with bipolar motors being more efficient. Consequently the steady-state force, *F*_*ss*_, is determined by forces exerted in parallel bundles and is independent of the bundle polarity. **(B)** Shows *F*_*ss*_ as a function of the fraction of bipolar cross-bridges for varying numbers of MTs. The same stall force is used for both unipolar and bipolar motors and *F*_*ss*_ is found to be proportional to λ*f*_*s*_ (see Section 3.3). The MT length in all curves of **(A,B)** is 10 μm, except for the dashed blue curve in **(B)** which is for l = 5 μm. Note the sharp drop in bundle force as the bipolar motor fraction surpasses the value χ_*bp*_ ≈ 0.8 (indicated by a black arrow). Other parameters used in these calculations are: *v*_0_ = 1 μm/s, *L*_0_ = 50 μm, *k* = 10 pN μm, χ = 1.

Figure 2A illustrates the calculated evolution of *L*(*t*) and *F*(*t*) in mixed bundles of filaments that are cross-linked by different ensembles of motors. Blue curves correspond to bundles with unipolar cross-bridges only, purple curves to bundles with bipolar cross-bridges only, and black curves to bundles with a 50% mixture of unipolar and bipolar cross-bridges; solid, dashed and dotted curves respectively represent different polarity ratios, *N*_*R*_∕*N* = 1, 0.2, 0.5, of filaments in the bundle. Bundles comprising unipolar cross-bridges only (blue curves) are shown to exert the strongest steady state forces, and the addition of bipolar motors hinders force generation. Between parallel filaments, bipolar motors behave as transient cross-linkers and thereby hinder movement and force generation. Bundles cross-linked by these motors only, may only expand for a limited time until the bundles get sorted apart and motion stops (purple curves).

When interacting with anti-parallel filaments, both unipolar and bipolar motors have a tendency to sort the filaments apart (Zemel and Mogilner, 2009). The filaments are eventually sorted into two separate domains of parallel filaments, one comprising only right-oriented filaments, and the other only left-oriented filaments, with a transition zone between them. The inset in Figure 2A provides a quantification of the dynamics of sorting. An order parameter, $S\left(t\right)={\left(N\;L\left(t\right)\right)}^{-1}\int_{0}^{L\left(t\right)}|n_{R}\left(x,t\right)-n_{L}\left(x,t\right)|dx$, has been defined to average the local polarity of the bundle (namely, the local difference between the number of right- and left-oriented filaments, |*n*_*R*_(*x, t*)−*n*_*L*_(*x, t*)|∕*N*) over the bundle length at any given time; *S* = 1 corresponds to a bundle that is locally parallel across its length, and *S* = 0 to a bundle that is locally mixed everywhere. The panel shows that sorting occurs on a time scale of ~10 s and that bipolar motors sort the filaments faster. Consequently, only the initial expansion dynamics depend on the bundle polarity, *N*_*R*_∕*N*, while the steady-state force and length are polarity-ratio independent (compare dotted, dashed and solid curves in Figure 2A). We find that the time to reach the stationary state for the (50%) orientationally-mixed bundles (dotted curves) is longer than for the more polar ones. In the mixed bundles, the initial sorting of filaments reduces the force that can be generated against the boundary. This is because filaments that undergo sorting move faster and contribute less to force generation; as a consequence the bundle expands more slowly^1^.

Figure 2B shows the calculated steady-state force, *F*_*ss*_, for bundles with varying fractions of bipolar cross-bridges. We find that the exerted steady-state force is a monotonically decreasing function of the fraction of bipolar cross-bridges. Interestingly, as soon as the fraction of bipolar cross-bridges surpasses a critical value, the force exerted by the bundle sharply decreases to zero (this is more clearly seen with the dashed-blue curve plotted for bundles with 5 μm long filaments in which the sharp change occurs at a smaller value of χ_*bp*_ ≈ 0.8). The sharpness of this transition and the critical value of χ_*bp*_ increase with the number of filaments and their length. The sharp transition and the dependence on system size are reminiscent of a percolation transition occurring in the bundle. Since bipolar motors do not exert sliding forces between parallel filaments (Equation 5), but rather behave as passive frictional elements between them, they are unable to sustain long-term stresses in the bundle. Thus, a system with a high fraction of bipolar cross-bridges and low fraction of (orientationally correlated) unipolar cross-bridges may become disconnected from one side to the other and long-term forces cannot be generated in such bundles. For a sufficiently large number of filaments, this so-called percolation transition occurs at a critical fraction of active overlaps in the bundle that depends on the density of filaments and their length, as discussed next.

### 3.2. Percolation transition dictates a motor-density threshold for force generation

**Figure 3:**
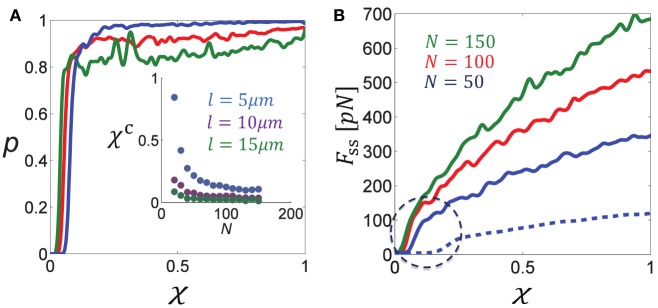
**Percolation transition in cross-linked bundles of filaments. (A)** Shows the percolation probability, *p* (of a bundle of fixed length *L*≈*L*_0_ to be interconnected from left to right), as a function of the fraction of active cross-links, χ, for different numbers of MTs (color coding is indicated in **B**). The percolation probability is seen to sharply increase to ~1 when a critical fraction χ^*c*^ is surpassed. The inset figure shows that χ^*c*^ is a decreasing function of the filament density, *N*∕*L* and length, *l*. **(B)** Shows the steady-state force, *F*_*ss*_, as a function of χ for different *N*. Solid curves correspond to l = 10 μm and the dashed blue curve to l = 5 μm. Note the sharp increase in *F*_*ss*_ when χ surpasses the percolation threshold, χ^*c*^, that is found in **(A)**. Parameters used in these calculations: *L*_0_ = 50 μm, λ*f*_*s*_ = 10 pN/μm, *k* = 10 pN/μm.

To demonstrate that the sharp changes in force generation indeed result from a percolation transition we have directly calculated the percolation probability, *p*, in bundles containing unipolar motors only and systematically varied the connectivity parameter, χ (see Figure 3). *p* is defined as the probability for the bundle to be percolated, namely, that at least one route of interconnected MTs transverses the bundle from left to right and allows the transmission of force to the boundaries. To calculate this probability numerous bundle configurations were generated with fixed numbers of MTs of same length, *l*, and fixed connectivity fraction, χ (the number of overlaps cross-linked by motors divided by the total number of overlaps between neighboring filaments); we also used a relatively stiff spring to prevent significant bundle expansion and thereby kept the density of filaments, *N*∕*L*, fairly constant. For each configuration we determined whether the bundle was percolated or not. *p* was then determined as the number of percolated bundles divided by the total number of configurations generated, and we investigated it as a function of *N*∕*L*, *l*, and χ.

Figure 3A shows the percolation probability, *p*, as function of the connectivity fraction, χ, for varying MT densities *N*∕*L*. Figure 3B shows the calculated force exerted by the bundle. Since expansion was minor, the calculated force was also the steady-state force. As expected, the point at which the force increases sharply with χ is the point where the percolation probability increases sharply to 1. We denote this percolation threshold by χ^*c*^. The inset figure in Figure 3A shows that χ^*c*^ is a decreasing function of the MT density and MT length. This result is expected since the longer the filaments, or the higher their density, the more likely it is to find an interconnected route of filaments that transverses the bundle. Another expected behavior is that the percolation transition becomes sharper and resembles a phase transition as the system size (number of filaments) increases (Stauffer and Aharony, 1992). The percolation threshold is a structural property of the bundle reflecting the hexagonal organization of the filaments in the *y* − *z* plane and their axial spread along the *x*-axis. We find that χ^*c*^ varies from ~0.08 for 50 filaments to ~0.04 for 150 filaments in case that l = 10 μm, and correspondingly between 0.1 and 0.2 when l = 5 μm. For comparison, the bond-percolation threshold is 0.34 for a two-dimensional triangular lattice and 0.25 for a three-dimensional cubic lattice (Stauffer and Aharony, 1992).

Interestingly, the percolation thresholds calculated in Figure 3A are consistent with those found in the previous section when the fraction of bipolar cross-bridges was varied systematically, indicating that the sharp decrease in force with χ_*bp*_ = 1 − χ_*up*_ resulted from a percolation transition. Another consequence of the percolation transition has been reported in our previous investigation of circularly-closed ringed bundles. It has been demonstrated that beyond a threshold level of bundle connectivity the induced velocity of the filaments sharply drops down due to interference of motor activity (Zemel and Mogilner, 2009).

The demonstration that interconnected bundles of MTs may undergo a sharp transition in their capacity to generate a force with variations of unipolar motor density may have important implications for neurite initiation and growth. During neuronal development force thresholds must be met to permit initial neurite extension and axon specification (Heidemann and Buxbaum, 1993; Chada et al., 1997; Fass and Odde, 2003). Following initiation, neurites undergo rapid growth and retraction cycles. Subsequently, one neurite starts rapid growth and becomes the axon. We suggest here that availability of unipolar motors at the cell periphery might be one of those limiting factors; once it surpasses a critical threshold, MT bundles in that region can become percolated and neurite initiation may proceed.

### 3.3. Scaling laws of force generation in bundles with a fixed number of filaments

**Figure 4:**
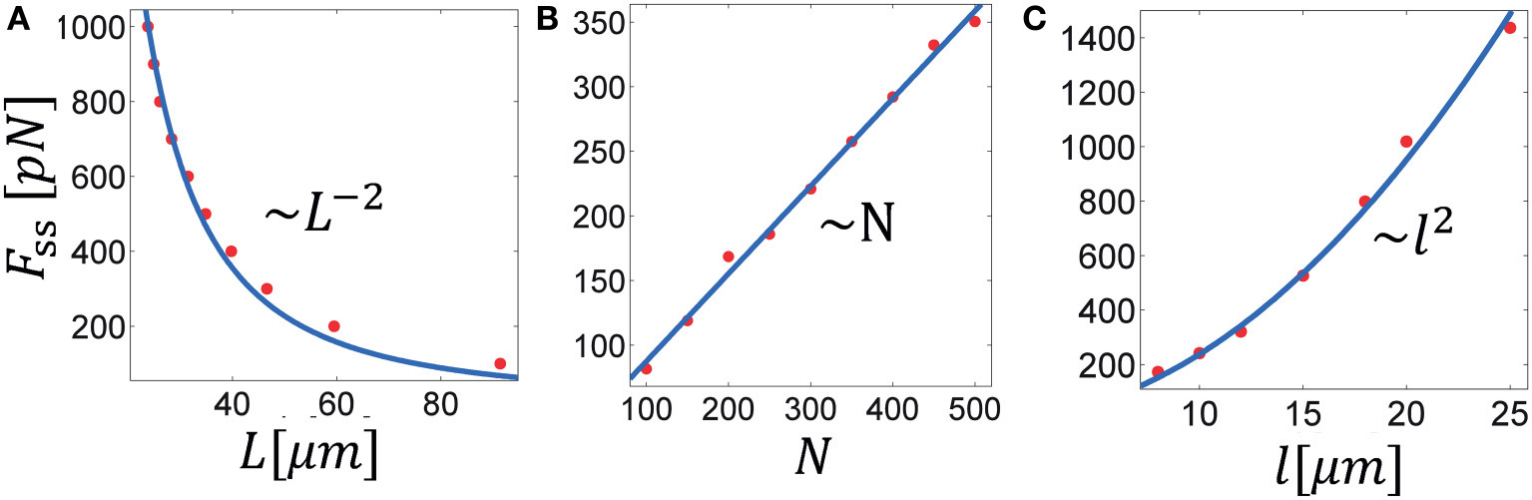
**Scaling laws in MT bundle force generation**. The three panels show the steady-state force as function of: **(A)** the steady-state bundle length, **(B)** the number of MTs, and **(C)** the MT length. Solid lines represent best fits of the denoted scaling law to the calculated data. These results are summarized in Equation (10), see text. Parameters used in the calculation: λ*f*_*s*_ = 10 pN/μm, χ_*up*_ = 1; in **(A,C)**: *N* = 300; in **(A,B)**: *l* = 10 μm.

Another important characterization of cytoskeleton-motor bundles is the relation between the bundle geometric characteristics, diameter and length, and the force that it generates. We concentrate here on forces generated in bundles with a fixed number of filaments; growth arising from an influx of filaments will be discussed in the next section. Because the MTs overlap, the generated force not only depends on the number of filaments in a cross section, but also on the mean overlap length between them. We shall therefore also reveal how the force/length relation depends on the MT length, *l*, and the motor connectivity factor, χ. The relationships discussed here pertain to the steady-state where bundle length and force are stationary. For clarity, we omit the subscript indication of the steady-state and denote the steady-state force and length by *F* and *L*, respectively. The following simulations have been carried out: (i) Bundles with a fixed number of MTs, *N*, were allowed to expand against an opposing spring with varying degrees of stiffness and the resulting steady state length and force were measured. We performed these calculations in bundles comprising MTs of different lengths and with variable degrees of the motor connectivity parameter χ (Figure 4A). (ii) We used a stiff spring to prevent changes of bundle length and carried out simulations with varied *N* (Figure 4B) or *l* (Figure 4C). In a given simulation all MTs had the same length. Since bundle polarity showed only little effect on the dynamics of bundle expansion and had no effect on the steady-state force, all simulations were carried out with polar bundles. Our results can be summarized via the following scaling relation:

(10) $$F\left(L,N\right)/(\lambda f_{s})\sim \sqrt{\chi }\;l^{2}N/L^{2}$$

with the geometric relation:

(11) $$L\;d^{2}\sim N\;l$$

where *d* is the bundle diameter. The scaling expressed in Equation (10) is demonstrated in Figure 4, showing *F* separately as function of *L*, *N*, and *l*. The scaling of *F* with $\sqrt{\chi }$ is seen in Figure 3B for χ > χ^*c*^. To explain these dependencies we first note that for any given cross-section in the bundle the force scales as, $F\sim \lambda \;{\bar{l}}_{ov}\;\bar{m}$, where $\bar{m}$ is the mean number of MTs in a cross section, $\lambda {\bar{l}}_{ov}$ is the mean number of motors interacting with a given filament and ${\bar{l}}_{ov}$ is the total average length of overlap per filament. We find in our simulations that ${\bar{l}}_{ov}\sim l∕L$. The inverse dependence on bundle length reflects the increase in number of interactions per filament with bundle compression; the square root dependence on χ follows because for a given cross section with *m* filaments around a given filament, ~ *m*^2^ is the total number of pair interactions and χ*m*^2^ is the fraction of those occupied by motors; hence only $\sqrt{\chi }m$ interact with one given filament in that cross section. Combining these relations, using Equation (11) and noting that $\bar{m}\sim d^{2}$ one arrives at the scaling in Equation (10).

### 3.4. Inward microtubule flux and bundle growth against a load

**Figure 5:**
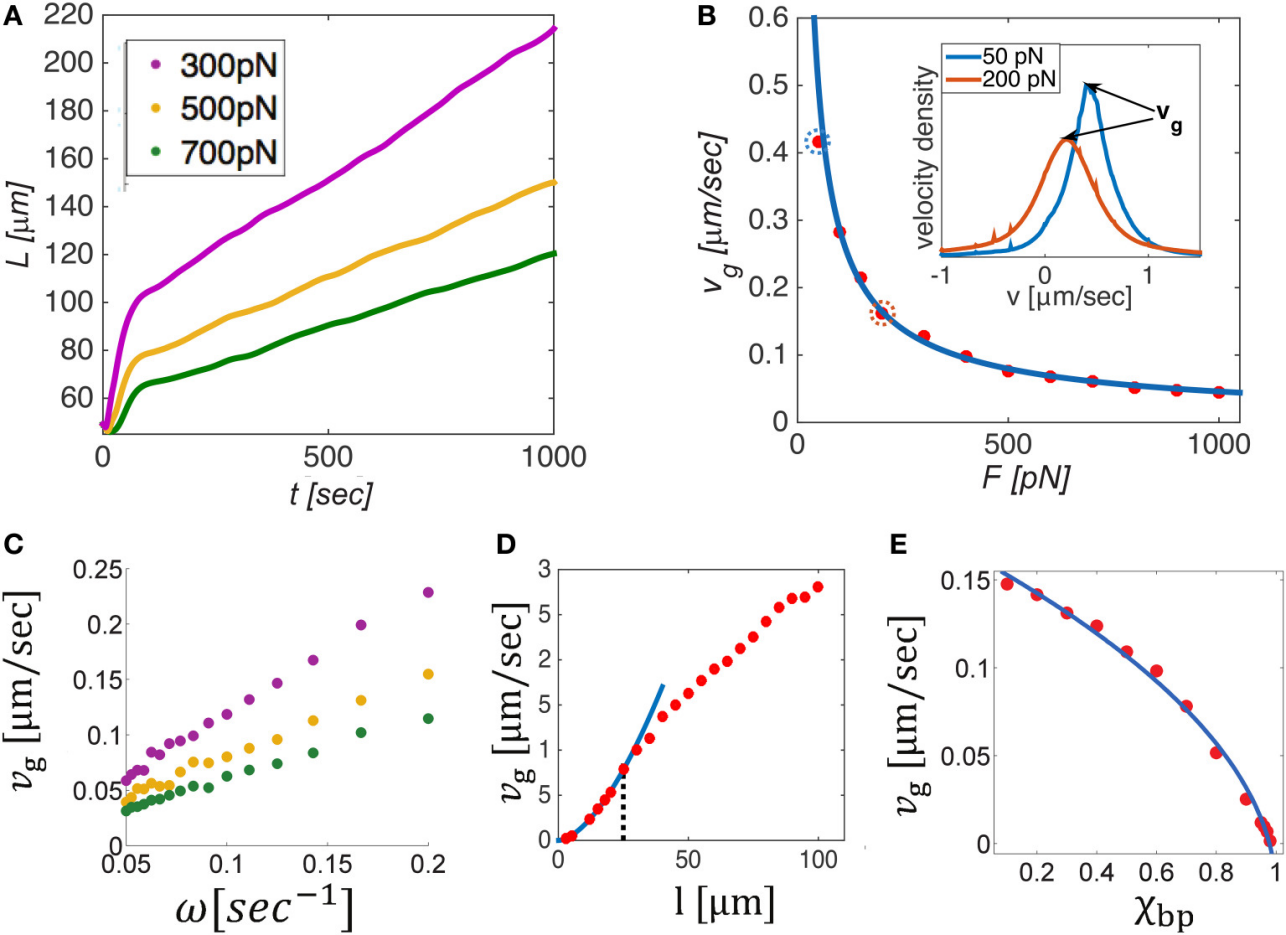
**Dynamics and mechanics of bundle growth against a constant opposing load when a constant supply of MTs is added on the left boundary at a fixed frequency, ω. (A)** Shows the evolution of bundle length, *L*(*t*), for different levels of opposing load. Beyond a short equilibration period, the bundles grow linearly in time and a constant velocity *v*_*g*_ can be defined. The steady state growth rate, *v*_*g*_, is shown in **(B–E)** as a function of the opposing load, *F*, the influx frequency ω, the MT length, *l*, and the bipolar cross-bridge fraction χ_*bp*_. Solid lines in panels B and E provide best fits to the simulation data. *v*_*g*_ is found to decrease with the load as *F*^−0.8^ **(B)**, and to linearly scale with ω **(C)**. The inset in **(B)** shows velocity distributions of the filaments for *F* = 50 pN and *F* = 200 pN (circled dots in the main panel). The peak velocity equals *v*_*g*_, implying that most filaments move as one mass with the growth velocity. The dependence of *v*_*g*_ on filament length **(D)** is biphasic: changing from $v_{g}\sim l^{1.8}$ for short MTs (*l* < 25 μm, blue curve) to $v_{g}\sim l^{0.8}$ for longer MTs; the transition point (dotted black line) depends on the stall force of the motors, see text. Interestingly, *v*_*g*_ is seen to rise above the motor free velocity, *v*_0_ = 1 μm/s, illustrating that the bundle can grow much faster than individual motors are able to move. Increasing the amount of bipolar motors in the system decreases the growth velocity severely and finally halts growth for χ_*bp*_ = 1. Unless otherwise mentioned we used *N* = 100, *L*_0_ = 50 μm, ω = 0.1 s^−1^, *l* = 10 μm, χ_*up*_ = 1, *v*_0_ = 1 μm/s, and λ*f*_*s*_ = 10 pN/μm. In **(D)**, *L*_0_ = 120 μm to allow longer filaments to be explored. In **(D,E)**, we used *F* = 200 pN.

We now consider the growth of MT bundles in the presence of a constant influx of filaments. This is relevant for growing neurites that extend over considerable distances and thus require a constant supply of new MTs. Experiments have demonstrated that the application of a pulling load on the cell membrane of neurons may initiate axon growth if a threshold level of force is applied. Moreover, this growth rate has been shown to linearly increase with the applied load (Zheng et al., 1991; Chada et al., 1997; Fass and Odde, 2003). Motivated by these results we have carried out the following simulations to investigate the properties of bundle growth under varying levels of *opposing* load when a constant supply of filaments is added per unit time. MT bundles were prepared with a starting number of *N*_0_ = 100 filaments and initial length *L*_0_ and new filaments of length *l* were added at a constant frequency, ω, on the left hand side of the bundle; the bundles were allowed to expand against a fixed load, *F* on the right boundary and a stiff spring on the left boundary. The simulations were carried out with varying levels of *F* (Figure 5B), and ω (Figure 5C); we also investigated the effects of the MT length, *l* (Figure 5D), and the fraction of bipolar cross-bridges in the bundle χ_*bp*_ (Figure 5E), assuming that all MT overlaps are occupied, either by unipolar motors or by bipolar motors χ_*bp*_ = 1 − χ_*up*_.

The growth dynamics for varying load levels are shown in Figure 5A. After a short period during which the filaments adjust the overlap between them against the load, growth continues linearly in time at a constant speed, *v*_*g*_ = (*dL*∕*dt*)_*ss*_. At this state, both the mean number of filaments in a cross-section, $\bar{m}$, and the mean overlap length between filaments remain fixed. Figures 5B–E, respectively show how *v*_*g*_ depends on the applied load, *F*, the inward flux rate, ω, the microtubule length *l* and the fraction of bipolar cross-bridges in the bundle, χ_*bp*_. We find that the growth rate scales as: $v_{g}\sim \omega \sqrt{\chi_{up}}\;l^{\alpha }∕F$, where α varies from α = 1.8 for short MTs to α = 0.8 for long MTs at some intermediate MT length (here, *l* ≈ 25 μm) that decreases/increases with decreasing/increasing stall force of the motors.

These dependencies can be explained based on the geometric relation, $L=N\;l∕\bar{m}$, expressed in Equation (11), where $\bar{m}\sim d^{2}$. Taking the time derivative of the equation above while keeping $\bar{m}$ fixed results in the following intuitive expression for the steady-state bundle growth rate *v*_*g*_:

(12) $$v_{g}=\frac{\omega \;l}{\bar{m}}$$

where ω = *dN*∕*dt*. The added MT mass per unit time, ω *l*, distributes over the bundle cross section $\bar{m}$, to produce a unit change in bundle length. Interestingly, $\bar{m}$ turns out to be proportional to *F*, which means that during growth the bundle adjusts its cross-section to match the number of filaments that share the load. Moreover, we find that the proportionality factor, $F∕\bar{m}$, scales linearly with the motor density (per unit filament length, λ) and stall force of the motors, and as the square root of the unipolar cross-bridge fraction: $F∕\bar{m}\sim \lambda \;f_{s}\sqrt{\chi_{up}}$. The $F∕\bar{m}$ ratio reflects the number of motors that share the load per filament in a cross section. The square root dependence on χ_*up*_ arises because the number of *active overlaps* driving a given filament scales in this way, as previously explained in Section 3.3.

The proportionality factor between *F* and $\bar{m}$ depends in a non-trivial way on the MT length. For short MTs, $\bar{m}∕F$ decreases with *l* and for long MTs it increases smoothly with *l* (not shown). This reflects the manner by which the filaments in the bundle adjust their interactions in response to the load. There are principally two ways in which they can do so: (i) by increasing the average overlap between pairs of filaments, and (ii) by increasing the number of filaments in a cross-section, $\bar{m}$. For short enough MTs, only the latter choice is possible, hence $\bar{m}$ decreases with *l*. For long MTs, (i) and (ii) provide two degrees of freedom for achieving force balance, hence $\bar{m}$ depends weakly on *l*. This behavior predicts an interesting dependence of bundle width on the MT length. In addition, it provides an explanation for the sigmoidal dependence of the growth rate, *v*_*g*_, on *l*, which shows an approximately quadratic dependence for short MTs, followed by a (nearly) linear dependence for long MTs, as shown in Figure 5D. The transition between these two scaling regimes depends on the stall force of the motors. The larger the stall force the better the filaments can sustain the load without increasing the number of filaments in a cross section, $\bar{m}$. Consequently, the transition point shifts to lower values of *l* when λ*f*_*s*_ is increased.

Summarizing these considerations we conclude:

(13) $$v_{g}\sim \frac{\lambda f_{s}\;\sqrt{\chi_{up}}\;\omega l^{\alpha }}{F}$$

where α ≈ 1.8 for short MTs and α ≈ 0.8 for longer MTs and the transition occurs at some intermediate MT length that decreases with the stall force of the motors.

The inset of Figure 5B show the velocity distribution of filaments in the bundle for two cases of opposing force (50 and 200 pN). Notably, most filaments are found to move with the growth velocity, *v*_*g*_. This implies that during steady-state growth the relative velocity between most filaments is zero. Although some filaments do continue to perform back and forth movements along the bundle length, the majority of them remain stationary with respect to each other and are collectively pushed as one mass at a constant velocity *v*_*g*_. In this situation, most motors are stationary, exerting their maximal stall-force *f*_*s*_. A remarkable consequence of this behavior is that the growth velocity of the bundle is independent of the free velocity of the motors, despite the fact that the entire motion is driven by motor activity only. Another important prediction is that the velocity distribution of filaments becomes wider when the opposing force is increased. The reason is that under conditions of larger force, the number of filaments in a cross-section is larger and this increases the diversity of possible interactions between filaments. As a consequence a wider range of filament velocities is found.

**Figure 6:**
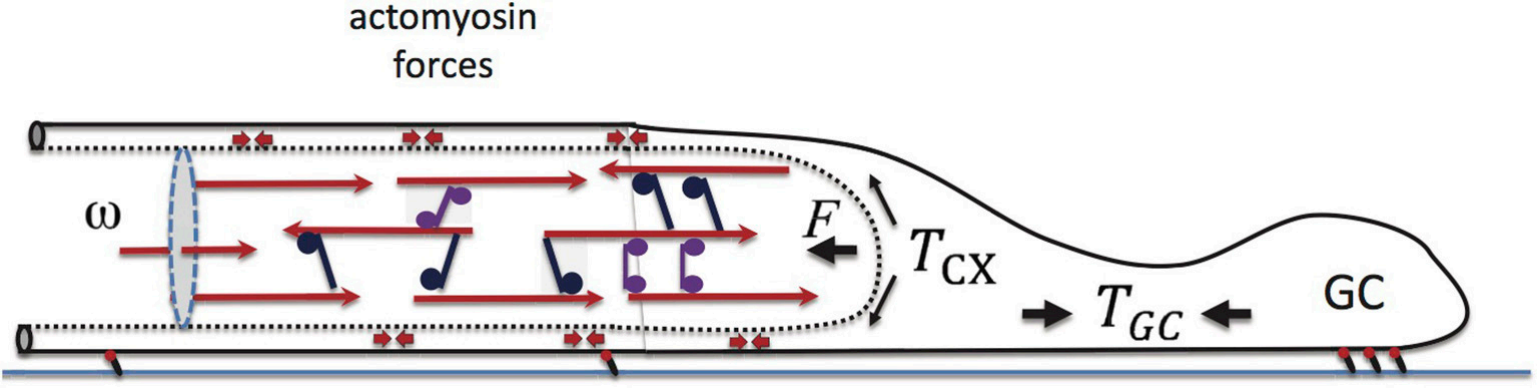
**Elements of force balance during neurite growth**. Our major focus is on the motor-cross-linked MT bundle at the neurite core. The opposing force on the neurite, *F*, arises from actively generated actomyosin tension, *T*_*cx*_, in the neurite cortex, and is biased by the GC-generated tension, *T*_*gc*_. Actomyosin, force dipoles embedded in the cortex are illustrated with small double headed arrows. Also shown is the inward flux, ω, which is found to play a primary role in determining the neurite growth rate, *v*_*g*_.

These results are consistent with experimental observations on neural growth. It is frequently reported that MT filaments in axons and dendrites are mostly stationary and that growth is in the range of 1 μm/min, an order of magnitude slower than the free velocity of typical motor proteins (Howard, 2001; Suter and Miller, 2011). According to our calculations the growth velocity is primarily dictated by the inward flux of filaments and the load acting on the bundle. This is consistent with the experimentally determined linear dependence of the growth rate on an external pulling load. This is because external pulling of the neurite tip relieves the opposing force acting on the growing MT bundle (see Figure 6). In a restricted range of loads, the scaling of *v*_*g*_ with *F* appears linear (see Figure 5B for *F* ≳ 200 pN) which might explain the reported experimental observations (Zheng et al., 1991; Chada et al., 1997; Fass and Odde, 2003). The threshold level of force (not to confuse with the percolation transition in Section 3.2) for axon initiation may relate to a number of factors, e.g., to the densities of MTs and/or the unipolar motors at the cell periphery, which limit the force that emerging bundles can spontaneously exert against the restoring forces in the plasma membrane and the underlying actin cytoskeleton.

## 4. Discussion

In this manuscript we focused on a major cytoskeleton structure contributing to the force balance of emerging neuronal processes—the heavily cross-linked MT bundle(s) in the neurite core. While there is ample evidence that both the MT filaments and the motor proteins associated with them are essential for axon elongation (Suter and Miller, 2011; Dehmelt, 2014), there is limited understanding of how these 1D molecular assemblies function. The major limitation in investigating these structures is their small size and their highly dense and compact architecture, which impedes quantitative imaging and analysis of their structure. Furthermore, there is also limited theoretical understanding of how assemblies of molecular motors and cytoskeletal filaments function collectively. We here used coarse-grained computer simulations to investigate the dynamics and force generation properties of such bundles. Our work, although presented in the context of axon initiation and growth, is relevant also for many other cellular processes in which such bundles play a key role, such as mitosis, blood platelet formation, and apoptosis (Scholey et al., 2003; Patel et al., 2005). The approach we took is intermediate between detailed computer simulations (Nédélec, 2002; Mogilner et al., 2006; Paul et al., 2009; Kim, 2014; Bidone et al., 2015; Ward et al., 2015), in which the motion and power-stroke dynamics of all individual motors are accounted for explicitly, and continuum approaches (Kruse and Julicher, 2000; Kruse et al., 2003; Liverpool and Marchetti, 2003; Ziebert and Zimmermann, 2005), which account for the motor-induced fluxes of filaments in an average manner, and in which only pairs of interacting filaments are taken into consideration. Our approach enabled us to investigate the dynamics exhibited by heavily cross-linked bundles of filaments. Rather than representing the motors individually, we used the force-velocity relationships that characterize their motion, and we did so simultaneously for all overlapping regions between filaments that are cross-linked by motors. This approach allows to simulate the dynamics of such bundles on an hours time scale (which is relevant for neurite growth) and to average over ensembles of trajectories in order to reveal some of their generic properties.

We first used our simulations to investigate how the motor type, unipolar or bipolar, the polarity ratio of the filaments, and the bundle connectivity affect the capacity of these bundles to exert a force. We found that both types of motors efficiently sort out oppositely-oriented filaments. Within seconds to minutes, the bundles get sorted and the dynamics become dominated by interactions between parallel filaments. Thus, the polarity ratio of the bundle has only a marginal effect on the dynamics of bundle expansion and no effect on its force generation capacity in the steady-state. Furthermore, our simulations robustly showed that only unipolar motors can provide the driving force for bundle expansion while the presence of bipolar motors impedes it. This result is expected since bipolar motors are unable to exert a force between parallel filaments, see Equation (5) and (Kapitein et al., 2005). In addition, the presence of bipolar motors in the bundle competes with the binding of unipolar motors to the filaments, and due to the reduced occupancy of the latter they slow down bundle expansion and weaken overall force generation. This is shown in Figure 2B, where the effect of χ_*bp*_ is demonstrated.

Importantly, we conclude that unipolar motors can drive bundle expansion only if the motors bind the filaments in an orientationally coordinated fashion (Figure 2B). If these motors randomly bind the filaments they exert a similar inhibitory effect on bundle expansion as bipolar motors do (cf. Equations 4 and 5). Our calculations may thus provide insight into the orientational organization and type of motors responsible for force generation in the dense MT bundles of neurites. Such information on the binding of dynein and kinesin-1 motors in the MT bundles of neurites or other cellular structures is, however, still lacking. Evidence for correlated binding of dynein to MTs has been reported in some *in vitro* studies (Haimo and Rosenbaum, 1981; Haimo and Fenton, 1984). Orientational correlation in unipolar motor binding to filaments may arise from steric or specific interactions between the motors and between the motors and the filaments. We hypothesize that it may also arise from a force-dependent binding rate of the motors. Because oppositely oriented motors between parallel filaments hinder each others' motion, the forces exerted by the motors are enhanced; this may cause the motors to detach from the filaments and to rebind with higher affinity in a correlated fashion. To test this hypothesis, we carried out preliminary calculations using the Cytosim simulation package [46], which allows to track the dynamics of individual molecular motors in small clusters of filaments. Our calculations indicate that force-dependent detachment rates of motors indeed lead to their spontaneous organization in a correlated fashion between the filaments.

Our conclusions are consistent with experiments highlighting the role of the unipolar motors, cytoplasmic dynein (Ahmad et al., 2000; Roossien et al., 2014) and kinesin-1 (Lu et al., 2013, 2015) in neurite initiation and growth, as well as with studies on kinesin-5, a bipolar motor, that has been shown to have inhibitory effects on neurite growth (Haque et al., 2004; Myers and Baas, 2007; Falnikar et al., 2011). Kinesin-5 has been suggested to play an important role in steering the motion of the GC. In that region, kinesin-5, has been shown to prevent MTs from entering filopodia and impede the movement of the filaments they connect to, thus causing other MT bundles to forcefully orient the GC (Nadar et al., 2008).

We have furthermore demonstrated a sharp dependence of bundle force generation on the motor connectivity parameter, χ (Figure 3A), and equivalently, on the fraction of unipolar cross-bridges, χ_*up*_, in a mixture of unipolar and bipolar motors. Below a critical value, χ^*c*^, the fraction of overlapping MTs that are cross-linked by motors is not sufficient to form a percolated bundle; hence forces cannot be transmitted across the bundle to extend it forward. The likelihood of the bundle to be percolated drops sharply below χ^*c*^ (Figure 3B). This behavior, known as a percolation transition (Stauffer and Aharony, 1994), may play an important role in neurite initiation and axon specification. It has been shown that prior to axon specification, kinesin-1 (a unipolar motor) accumulates at the tip of the emerging neurite that eventually becomes the axon, Jacobson et al. (2006); it is absent in the tips of the other neurites that grow slower and later turn into dendrites. It is tempting to speculate that the sudden rise in kinesin-1 concentration at the tip of the future axon enables the MT-connectivity to surpass the percolation threshold necessary for the neurite to expand against the mechanical barrier of the actomyosin cortex, and thereby trigger a rapid growth phase that eventually leads to axon specification.

Variations in χ_*up*_ may arise due to the presence of bipolar motors and other MT-associated proteins (MAPs) which may compete with unipolar motor binding. In addition, variations in χ_*up*_ may arise due to the effects of MT binding proteins on the inter-filament spacing. MAPs vary in size, ranging from 80kDa (tau protein) to 200kDa (MAP2). Larger MAPs have been shown to increase the spacing between MT filaments in neurites (Chen et al., 1992; Mukhopadhyay and Hoh, 2001). Tau is enriched in axons, while MAP2 is mostly expressed in dendrites (Bernhardt and Matus, 1984; Kosik and Finch, 1987). Thus, axons have much shorter inter-filament spacing (~25 nm) than dendrites (~60 nm) (Chen et al., 1992). Small spacing between filaments, through enrichment of tau over MAP2, can thus facilitate the cross-linking of MTs by motors and cause χ to surpass the percolation threshold χ^*c*^ and thereby facilitate axon elongation. This might also explain how Tau contributes to inducing axon-like structures in Sf9 cells (Baas et al., 1991).

Our simulations allowed us to reveal generic scaling laws for the dependence of MT bundle force on the bundle length. One of the factors that strongly influences this dependence is the MT length, *l*. We find that for bundles that expand with a fixed number of filaments, *F* ~ *l*^2^. This dependency may be of importance during axon initiation and regeneration. Stabilization of MTs in neurites of primary cultures is known to precede cell polarization and axon growth. Furthermore, fast MT polymerization correlates with faster outgrowth (Baas and Ahmad, 1993; Witte et al., 2008; Lu et al., 2013). According to our predictions, the force exerted by bundles with long and stable MTs increases quadratically with the MT length, thus selective stabilization in one neurite could lead to enhanced neurite outgrowth and axon formation. These results may shed light on the striking recent demonstrations that the MT stabilizing drugs Taxol (Hellal et al., 2011) and epothilone B (Ruschel et al., 2015) may promote axon regeneration after spinal cord injury.

Finally, in the last results section, we considered the growth of MT bundles when a constant supply of MTs is added to the bundle per unit time; this is relevant for neurites in their growth phase (after initiation). The quantity ω *l* in Equation (12) dictates the rate of (net) MT mass addition into the bundle. During growth, this mass can either distribute across the bundle cross-section or contribute to elongation. We found that the bundles maintain a uniform number of filaments per cross-section, $\langle m\left(x\right)\rangle \equiv \bar{m}$, along their length (with slight variations near the fixed left boundary). This number, which reflects the width of the bundle, is determined by the load acting on the bundle. We find that $\bar{m}$ does not alter during steady-state growth and that it scales linearly with the opposing force on the bundle, $\bar{m}\sim F$. Thus, a load acting on the bundle determines its width during steady-state growth. This is consistent with experiments that have shown that the number of MTs per cross-section in developing axons remains constant during different growth stages (Baas et al., 1989). Hence, the overall caliber of the MT bundle remains unchanged in pre-synaptic axons.

In Figure 6 we have schematically illustrated a few elements that are believed to play a major role in the force balance of growing neurites (Suter and Miller, 2011). The cross-linked MT bundles found in the neurite core act against an actomyosin-filled cortex, that in turn connects to the “towing” machinery of the GC. Within the cortex, myosin II motors generate a tensile load, *T*_*cx*_, whose contribution along the *x*-axis is *F*. GC motility and actomyosin forces in this cellular domain produce elastic tension, *T*_*gc*_, at the cell front. The larger *T*_*gc*_, the weaker is the load *F* on the bundle, and consequently the neurite can grow faster. Additionally, *F* can be reduced experimentally by external pulling. In that case, *T*_*gc*_ is replaced by the applied force and consequently *v*_*g*_ increases as observed experimentally (Zheng et al., 1991; Chada et al., 1997; Fass and Odde, 2003).

These conclusions may provide important insight into the different functions of the MT machinery in axons and dendrites. Our predictions may also be applicable to other systems in which MT bundles play a key role, such as in the mitotic spindle of dividing cells, or within the pro-platelet shafts emanating from megakaryocytes during platelet formation (Patel et al., 2005). *In vitro* studies, which allow control of the MT density, bundle size, MT length distribution, and motor type, will be invaluable to test the scaling laws described in this manuscript.

## Author contributions

AZ and MJ wrote the simulation code, MJ ran the calculations, MJ, KF, AZ designed the work and wrote the paper.

### Conflict of interest statement

The authors declare that the research was conducted in the absence of any commercial or financial relationships that could be construed as a potential conflict of interest.

## Footnotes

^1^Calculations are presented for equal stall force for the bipolar and unipolar motors. When the bipolar-motor stall force is increased, only the dynamics of bundle expansion is affected but there is no effect on the steady-state force; *F*_*ss*_ is dictated by the stall force of unipolar motors (see Section 3.3). Because bipolar motors sort the filaments faster, sorting occurs more quickly when their stall force exceeds that of the unipolar motors, but the time to reach the steady-state is prolonged due to the increase in the viscosity of the bundle.
